# Southmead Orchestral Society

**Published:** 1970-01

**Authors:** 


### Southmead Orchestral Society

This grandiloquent title abbreviates appropriately to S.O.S. and while not really in distress the Society would like to enlist the aid of any members of the profession or their friends, relatives or acquaintances interested in playing orchestral instruments, particularly strings.

The Society had its birth in 1963 in a Clifton drawing room when a handful of people collected to try to play some Bach Brandenburg concertos. The experience proved so enjoyable that they have kept it up ever since, later moving to a rather larger drawing room equipped with a harpsichord and then as more members were collected to the waiting room of Litfield House.

Two years ago by kind permission of Dr. Geoffrey Tovey the orchestra moved to its present home, the beautiful little hall in the Blood Transfusion Centre at Southmead Hospital. Now with 50 members on the books it meets monthly and rehearses, renders or otherwise mutilates a programme of popular Classics—usually an overture, a Symphony and either a suite or—when there are volunteers—a concerto. In the interval we eat a little and drink quite a lot, so as to find our form for the main work—the Symphony.

We have never given a concert and have no intention at present of ever doing so, even under the guise of collecting for a charity! Our activities are purely for the fun of the thing. We do each programme twice and then move on to the next; over the years, in this way, we hope to play all our favourite pieces.

About a dozen of the members are on the staff of Southmead Hospital, a number of others are medical and nursing colleagues, but the bulk of the orchestra is composed of a variety of musical enthusiasts, a few of whom are really expert and with whose help we occasionally rise to unexpected heights. Our main inspiration is of course our conductor David Gibbs who, besides lecturing in physics at the University, can play any instrument you care to name, and seems to find amusement in our occasional weird cacophonies.

The orchestra meets on the third Saturday of each month at 7.30 p.m. at Southmead Hospital. Each member is asked to make a small contribution (currently 5/-) at each meeting to cover the cost of music and refreshments.

The programme for 1969-70 season is as follows:

**September and October**

Glinka —Overture Russlan and Ludmilla
Beethoven —Romance for Violin
Dvorak —New World Symphony

**November and December**

Beethoven —Fidelio Overture
Mozart —Symphony No. 40
Bizet —Carmen Suite

**January and February**

Rossini —Scala di Seta, Overture
Beethoven —Symphony No. 8
Elgar —Suite "From the Bavarian Highlands" (with Chorus)

**March and April**

Mozart —Horn Concerto, K 495
Haydn —Symphony No. 102
Sibelius —Suite Karelia

**May and June**

Mozart —Seraglio Overture
Brahms —Symphony No. 1
Elgar —Pomp and Circumstance March No. 1

If anyone — particularly a string player — cares to join us please contact the secretary, Mr. Michael Wilson at Southmead Hospital.

